# The role of sphingosine-1-phosphate in the development and progression of Parkinson’s disease

**DOI:** 10.3389/fncel.2023.1288437

**Published:** 2023-12-21

**Authors:** Wang Wang, Yang Zhao, Guoxue Zhu

**Affiliations:** ^1^Department of Neurology, Nanjing Hospital of Chinese Medicine Affiliated to Nanjing University of Chinese Medicine, Nanjing University of Chinese Medicine, Nanjing, China; ^2^School of Medicine & Holistic Integrative Medicine, Nanjing University of Chinese Medicine, Nanjing, China

**Keywords:** Parkinson’s disease, sphingosine-1-phosphate, S1P metabolism, S1P modulators, cellular neuropathology

## Abstract

Parkinson’s disease (PD) could be viewed as a proteinopathy caused by changes in lipids, whereby modifications in lipid metabolism may lead to protein alterations, such as the accumulation of alpha-synuclein (α-syn), ultimately resulting in neurodegeneration. Although the loss of dopaminergic neurons in the substantia nigra is the major clinical manifestation of PD, the etiology of it is largely unknown. Increasing evidence has highlighted the important role of lipids in the pathophysiology of PD. Sphingosine-1-phosphate (S1P), a signaling lipid, has been suggested to have a potential association with the advancement and worsening of PD. Therefore, better understanding the mechanisms and regulatory proteins is of high interest. Most interestingly, S1P appears to be an important target to offers a new strategy for the diagnosis and treatment of PD. In this review, we first introduce the basic situation of S1P structure, function and regulation, with a special focus on the several pathways. We then briefly describe the regulation of S1P signaling pathway on cells and make a special focused on the cell growth, proliferation and apoptosis, etc. Finally, we discuss the function of S1P as potential therapeutic target to improve the clinical symptoms of PD, and even prevent the progression of the PD. In the context of PD, the functions of S1P modulators have been extensively elucidated. In conclusion, S1P modulators represent a novel and promising therapeutic principle and therapeutic method for PD. However, more research is required before these drugs can be considered as a standard treatment option for PD.

## 1 Introduction

Parkinson’s disease (PD), described by James Parkinson in his 1817 “An essay on the shaking palsy” (Samii et al., 2004), is characterized by the typical symptoms include tremor, rigidity and postural instability, and bradykinesia, associated with non-motor symptoms such as cognitive impairment, sense of smell, gastrointestinal symptoms due to the degenerative loss of dopaminergic neurons and the formation of Lewy bodies (Pajares et al., 2020). With the global population aging at an accelerating pace and the expected average life span continuing to rise, neurodegenerative disease like PD is gaining attention from the scientific community. According to statistics from the Global Burden of Disease Study, the number of PD case will rise to 13 million in 2040 that is reaching epidemic proportions (GBD 2016 Parkinson’s Disease Collaborators, 2018). The treatment of PD is focused on the drug therapy including levodopa, catechol-o-methyltransferase inhibitors (COMT), monoamine oxidase type B inhibitor (MAOB), etc. (Ogawa et al., 2022). At present, levodopa is the most effective means for the motor symptoms of PD. However, levodopa, a natural stimulant, cannot cross blood brain barrier. And beyond that, Surgical treatment (deep brain stimulation) is also an effective way to improve the symptoms of PD patients (Tao and Liang, 2015). Nevertheless, these all methods cannot prevent, slow, or reverse the progress of the degenerative death of DA neuron as well as cannot effectively alleviate symptoms. Therefore, the realization is growing that novel PD targets and novel therapies are of upmost importance (Van Bulck et al., 2019).

Sphingosine-1-phosphate (S1P), a bioactive lysophospholipid produced by sphingosine kinases, plays a critical role in the regulation of cell growth, differentiation, and apoptosis through binding to a family of five G protein-coupled receptors (S1PR1-5) (Al Gadban et al., 2012; Ye et al., 2022). S1P exhibit a serious of biological effects, including oxidative stress, autophagy, anti-inflammatory, anti-apoptosis and improved mitochondrial function, and has a neuroprotective effect on PD (Sun et al., 2022). In addition, S1P is essential for vascularization and brain development (Van Echten-Deckert et al., 2014). Simultaneously, S1P can protect against dopamine depletion, neuroinflammation, and PD-associated symptoms by uncoupling the S1P1R from Gi-protein (Hait and Maiti, 2017; Lee et al., 2022). Most impressively, some studies have illustrated that S1P might regulate microglial phagocytosis (Xue et al., 2022). Hence, developing further understanding about the role of S1P in PD development and progression is of great significance and urgency to promote the timely and accurately diagnosis of PD.

## 2 Search strategy

We utilized PubMed and Web of science platforms for English language articles on this research. Meanwhile, two Chinese databases (China National Knowledge Infrastructure Database and Wanfang Database) will be searched for Chinese language articles. “Parkinson disease,” “S1P,” “Sphingosine-1-phosphate,” “modulators” as the key words used in database and many other keywords relevant to every section. We also reviewed bookson Parkinson’s disease or movement disorders published in the same period. We reviewed selected references from articles retrieved by the initial search. The contents of this article are based on reviewed published work, our judgment, consultation with experts in the area of PD.

## 3 S1P pathway overview

**FIGURE 1:**
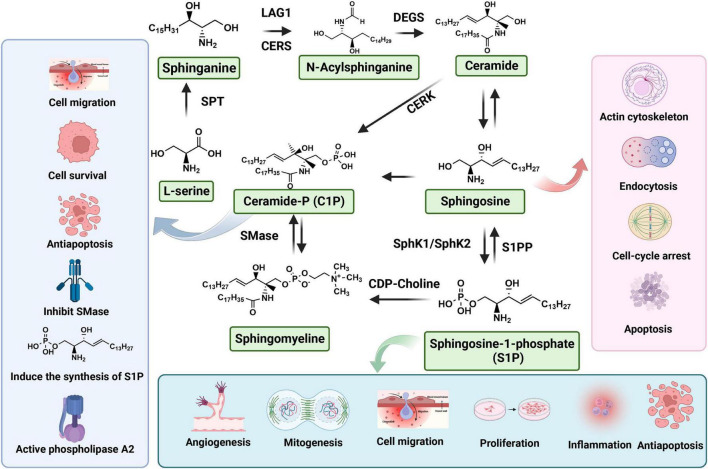
Biosynthesis and metabolism of sphingosine 1-phosphate (S1P).

Sphingolipids are considered to serve principally as structural components of cell membranes conferring unique signaling functions. S1P (molecular weight: 379.47 g/mol), also known as sphing-4-enine-1-phosphate, is classified as a small molecular weight lipid and bioactive sphingolipid metabolite. S1P is a compound with potent bioactive actions in sphingolipid metabolism, the calcium signaling pathway, and neuroactive ligand-receptor interaction (Atkinson et al., 2017). Furthermore, S1P can be generated from the phosphorylation of sphingosine and catalyzed by sphingosine kinases (SphK1 and SphK2) in blood platelet, erythrocyte and endotheliocyte (Figure 1). In cells, S1P is recognized by a family of G-protein coupled receptors (GPCRs, S1PR1-5), degraded by the S1P lyase (S1PL) and reversibly conversion to sphingosine through S1P phosphatases (S1PP) (Mitrofanova et al., 2019; Hii et al., 2020). S1PL is an intracellular enzyme, which is a key enzyme to regulate the degradation pathway of sphingomyelin, as well as an important pathway of sphingomyelin metabolism (Choi and Saba, 2019). Abnormal function of S1PL can lead to accumulation of sphingomyelin in S1P and its upstream, leading to organ dysfunction (Tang et al., 2021). In contrast to the SphKs activity, S1PP activity is catalyzing the dephosphorylation of S1P into sphingosine (Figure 1). After binding with S1P, S1PRs can conjugate with different heterotrimer G protein subunits to activate downstream cell signaling pathways, such as PI3K/Akt (Liu and Tie, 2019) and MAPK (Liu et al., 2022) signaling pathway, so as to play the biological role of S1P. The S1P receptor is a G-protein-coupled cell membrane receptor that specifically binds to S1P, leading to the regulation of various biological effects mediated by S1P. It exhibits a high affinity for S1P and plays a crucial role in mediating important biological functions. S1PR1-3 is widely distributed in the body and highly expressed in lymphocytes, astrocytes, oligodendrocytes, etc. Alternatively, S1PR1-3 mainly regulates immunity, nerve cell migration, embryonic development of cardiovascular system and nervous system, and also plays a certain role in endothelial barrier function and angiogenesis. In addition, S1PR5 is mainly expressed in oligodendrocytes, astrocytes and other cells of the nervous system, which mainly regulates the function of oligodendrocytes and participates in the migration of natural killer cells (Chun et al., 2021).

## 4 The regulation of S1P signaling pathway on cells

Sphingosine-1-phosphate is produced by SphKs in blood platelet, erythrocyte and endotheliocyte and regulated by lyase and phosphohydrolase. For instance, the activation of SphK1 by abundant cytokines can cause the translocation of SphK1 from the cytoplasm to the cytomembrane. This translocation ultimately leads to the production and release of S1P (Jolly et al., 2005; Nayak et al., 2010). Currently, our understanding of the biological significance of S1P primarily revolves around the following two aspects: (1) it functions as a secondary messenger within cells and directly impacts downstream targets, leading to the regulation of diverse biological effects; (2) it is released into the extracellular environment through autocrine and paracrine mechanisms and travels in the bloodstream by binding to apolipoprotein. This apolipoprotein then interacts with specific S1P receptors, which are G protein-coupled, resulting in the regulation of various biological effects downstream. SphKs, widely existed in mammals, are the key enzymes to catalyze sphingosine to S1P. SphKs is a protein with an apparent molecular mass of 49 kDa, which originally purified from rat kidney (Obinata and Hla, 2012). There are two subtypes in the body (SphK1 and SphK2) and encoded by genes located on chromosome 17 (17q25.2) and chromosome 19 (19q13.2), respectively (Kihara et al., 2006). Interestingly, there are two SphK isozymes that differ in their temporal, spatial distribution and different biochemical properties. SphK1 was found to be located predominantly in the cytoplasm, while SphK2 is mainly exist in the nucleus (Hait and Maiti, 2017). SphK2 compensates for SphK1 in keeping tissue S1P in the SphK1 deficient state. SphK1 and SphK2 are show different functions in apoptosis, immune cell responses, inflammation, and cell survival, etc. (Li et al., 2020).

### 4.1 Effects of S1P signaling pathway on cell growth and proliferation

The overexpression of Sphk1 can induce cell proliferation through promoting the G1 phase transition to S phase and reducing doubling time in the cell cycle to regulate DNA synthesis (Olivera et al., 1999; Igarashi et al., 2003). After blocking Sphk1, cell cycle can be lengthened or arrested in G1 phase, pro-apoptotic proteins can be up-regulation to activate intracellular apoptotic pathways. In addition, SphK1 not only plays an important role in cell cycle but also blood vessel formation in the nervous system and inflammatory response. In contrast, Sphk2, a nuclear protein, can suppresses cell proliferation, induces cell cycle arrest and enhances apoptosis thus result in inhibiting DNA synthesis (Igarashi et al., 2003; Weigert et al., 2010). The nuclear localization sequence mutations of Sphk2 eliminates its inhibitory effect on cellular growth. SphKs inhibition could be contribution to the alteration of the S1P/ceramide inhibitor. S1P levels in the brain are mainly regulated via Sphk1 actions, and a dysregulation of this S1P/Sphk1 signaling has been reconditioned in numerous CNS pathologies (Ayub et al., 2021).

### 4.2 Effects of S1P signaling pathway on cell apoptosis

Apoptosis, which is characterized by morphological features, is the primary cause of pathophysiological cell death and regulated by a serious of pro-apoptotic, anti-apoptotic, and cell growth signals (Suzuki et al., 2013) in physiologic and pathologic conditions (Elmore, 2007). In addition, it plays an important role in development, aging, and defense mechanism via immune system. Apoptosis is the primary target for anti-cancer research through two pathways: intrinsic mitochondrial-dependent pathway and the extrinsic death receptor-mediated pathway. Among of them, caspases are the dominant enzymes and synchronized apoptosis events act as a cysteine-dependent aspartate-directed proteases, such as caspase-8 mediates extrinsic pathway (Ashkenazi, 2015), while caspase-3 mediates intrinsic pathway (Loison et al., 2014).

**FIGURE 2:**
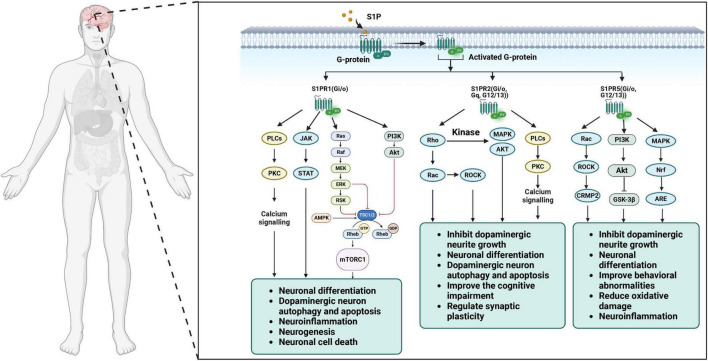
S1P receptor subtypes and cellular signaling pathways in the brain.

S1P acts as an intracellular second messenger to inhibit apoptosis by binding to the receptor S1PRs. The balance between ceramide and S1P, act as “sphingolipid rheostat “, plays a vital role in cell apoptosis (Harvald et al., 2015). S1P is produced via the expression of SphK1 and prevents apoptosis by activating signaling pathways involved in cell survival, including inositol-independent Ca^2+^ mobilization, ERK1/2, AKT and nuclear factors (Xia et al., 1999; Spiegel and Milstien, 2003; Figure 2; Table 1). S1P also inhibits apoptosis-related signal cascades (Figure 2; Table 1), such as cytochrome c release from mitochondria, activation of caspase, and activation of c-Jun N-terminal protein kinase (JNK) (Spiegel and Milstien, 2003). Interestingly, SphK2, the other isoform of the kinase, has the effect of pro-apoptotic via regulating cell cycle arrest and cell death, thus counteracting the positive effect of SphK1 on cell division. Furthermore, SphK2 is redirected to endoplasmic reticulum in the state of starvation (Igarashi et al., 2003). The re-localization of SphK2 illustrated that the pro-apoptotic role of it is due to the production of ceramide from external sources of sphingoid bases through the combined effects of SphK2 and lipid phosphohydrolases in the endoplasmic reticulum (Ding et al., 2007). In turn, up-regulation of SphK2 increases the conversion of sphingosine to ceramide, while up-regulation of SphK1 reduces it (Maceyka et al., 2005). Interestingly, S1P which is distributed in different intracellular pools might play different roles in metabolic and signaling pathways. The fact that mutation of the Bcl-2 homology-3 (BH3) primary reasons suppression of the SphK2 induced apoptosis. Ceramide, the converted product of S1P, coordinates programmed cell death via two pathways (type I and type II). Type I programmed cell death, apoptosis, is induced by elevated levels of ceramide, by activation of caspase-9 via inactivation of protein kinase B (PKB), activation of Bad via Ras and protein phosphatase 2A (PP2A), and activation of protein kinase Cζ, etc. (Ruvolo et al., 1998).

**TABLE 1:** Interaction of S1PR modulators with S1PR subtypes and their physiological consequences.

Receptor	Location	Signaling pathway	Agonists	Agents	Functions	References
S1PR1	Spleen Brain Heart Lung Adipose tissues Liver Thymus Kidney Skeletal muscle	Gi/o	FTY720 SEW2871 KRP203 W123	Fingolimod Siponimod Ozanimod Ponesimod	Neural cell migration/function Vasculature formation Endothelial barrier function Development of nervous systems Neuroprotection	Kunkel et al., 2013, Pyszko and Strosznajder, 2014; Anwar and Mehta, 2020; Chen et al., 2021.
S1PR2	Lung Heart Brain	Gi/o Gq G12/13	ND	ND	Endothelial barrier function Hearing and balance Neural cell migration/function Neuroprotection Blood-brain barrier	Kunkel et al., 2013; Karunakaran et al., 2019; Tang et al., 2020; Xiang et al., 2021.
S1PR3	Heart Lung Spleen Kidney Intestine	Gi/o Gq G12/13	FTY720	Fingolimod	Endothelial barrier function Hearing and balance Neural cell migration/function Neuroprotection	Ishii et al., 2001; Kunkel et al., 2013; Kihara et al., 2015.
S1PR4	Lymphoid tissues	Gi/o G12/13	FTY720	Fingolimod	Oligodendrocyte function Natural killer cell migration Neuroprotection	Gräler et al., 1998; Graeler and Goetzl, 2002; Alexander et al., 2013; Kunkel et al., 2013.
S1PR5	Spleen and white matter tracts of the CNS	Gi/o G12/13	FTY720	Fingolimod Siponimod Ozanimod	Oligodendrocyte function Natural killer cell migration Neuroprotection	Im et al., 2000; Malek et al., 2001; Jaillard et al., 2005; Kunkel et al., 2013.

### 4.3 Effects of S1P signaling pathway on cell migration

The S1P signaling pathway plays a crucial role in regulating cell migration. S1P is a bioactive lipid that acts as a potent extracellular signaling molecule, binding to specific cell surface receptors and initiating intracellular signaling cascades (Rykowski et al., 2023). Especially, it is a complex process that plays a vital role in multiple physiological function, such as regeneration, angiogenesis, cell mediated immune responses, as well as tumor progression (Chi et al., 2022). Cell migration is a complex process that can be influenced by a variety of factors, which can roughly be divided into the following two categories: the chemical factors and the mechanical factors. The influence of S1P on cell migration is mainly manifested in the following aspects: promotion of cell migration, modulation of cytoskeletal dynamics (Donati and Bruni, 2006), regulation of cell adhesion, involvement in chemotaxis, and regulation of barrier function.

Cell migration can be regulated by S1P through blinding to S1P receptor, which is closely related to cell movement. Simultaneously, it is regulated by platelet-derived growth factor (PDGF), which is an effective activator of SphKs. Interestingly, PDGF signaling is fundamental to the development of brain vasculature and involved in variety neurodegenerative disease, especially in PD (Li S. Y. et al., 2022). PDGF tyrosine kinase receptors transactivate S1P pathway by stimulating the production of SphK1 and S1P, which is essential for PDGF to regulate cell movement. Once activated inside the cell, the generated S1P can regulate cell movement using “inside-out signaling” through its cell surface receptors. Nevertheless, the effect of S1P on migration is complex and may depend on the S1PRs expressed on the cell surface. Nicholas et al. (2017) found that the blinding of S1PR1 and S1PR3 with S1P many stimulate cell migration, whereas S1PR2 has adverse action.

Sphingosine-1-phosphate acts as a signaling molecule that binds to specific receptors on the cell surface, known as S1P receptors. Upon binding, the bioactive sphingolipid S1P induces significant reorganization of the cytoskeleton in diverse cell systems, primarily through a set of distinct cell surface receptors known as S1P receptors. Notably, S1P-induced alterations in cell morphology and/or movement through cytoskeletal remodeling play a functional role in the cell-specific biological effects exerted by S1P in diverse cellular systems, primarily mediated by a group of distinct cell surface receptors called S1P receptors (Donati and Bruni, 2006). S1P signaling affects cell adhesion molecules, such as integrins, which mediate cell-substrate interactions. It can enhance integrin activation, leading to increased cell adhesion to the extracellular matrix and facilitating cell migration (Mendelson et al., 2014). Remodeling plays a functional role in inducing chemotaxis of primary fibroblasts by the sphingolipid S1P, primarily mediated by S1P receptors (Gil et al., 2010; Li Y. et al., 2022). In line with the migratory reaction, S1P induced the elongation of lamellipodia at the outer edge of human fibroblast cells and reorganized the cytoskeleton. In fibroblasts, the chemotactic response mediated by S1P was impaired upon downregulation of decapentaplegic family member 3 (Smad3) (Huang et al., 2022). Therefore, it suggests that the downstream signaling pathways involving Smad3 are necessary for S1P to properly function in maintaining barrier function (Zhang and Wang, 2023).

### 4.4 Effects of S1P signaling pathway on immune responses

The innate immune system is the basis of host survival and overall health, the first line of defense against initial invading organisms and environmental challenges, and the fundamental for maintaining tissue homeostasis (Baumruker and Prieschl, 2002; Liaskou et al., 2012). The overall survival of the host relies on its ability to identify and alleviate appropriate defense signals to eliminate infectious microorganisms (Liaskou et al., 2012). The human brain organ is considered an immune privileged organ due to the blood-brain barrier (Tan et al., 2020). These findings illustrate that innate and adaptive immune responses could be contributed to the central nervous system (CNS) in neurodegenerative disease, especially PD (Harms et al., 2021). The increased risk of PD is associated with some autoimmune diseases, suggesting that autoimmunity have an effect on the pathogenesis of PD (Tan et al., 2020). The balance and downstream signal regulation of S1P and sphingosine also play an important role in the immune system (Baumruker and Prieschl, 2002). Especially in the immune response, high-level sphingosine significantly inhibits the synthesis of leukotriene and the production of cytokine in response to antigen-induced immunoglobulin in mast cells (Prieschl et al., 1999). S1P is a bioactive lipid molecule that binds to S1P receptors, and triggers a series of intracellular signaling events. These events ultimately affect the behavior and function of immune cells. One of the primary effects of S1P signaling on immune responses is the regulation of lymphocyte trafficking (Tsai and Han, 2016). Lymphocytes, such as T cells and B cells, are key players in the immune system and are involved in various immune processes. S1P acts as a chemoattractant, guiding lymphocytes out of lymphoid organs, such as the thymus and lymph nodes, and into the bloodstream. This process, called lymphocyte egress, is crucial for immune cell distribution and surveillance throughout the body.

Moreover, S1P signaling influences the migration of immune cells to specific sites of inflammation or infection (Tian et al., 2022). When immune cells encounter tissue damage or infection, S1P gradients are established, attracting immune cells toward the affected area. This helps in the recruitment of immune cells to sites of injury or infection, enabling an effective immune response. S1P signaling also modulates the function of various immune cell types. For example, S1P has been shown to regulate the activation and proliferation of T cells (Romani et al., 2018). It can enhance T cell activation by promoting the secretion of cytokines and increasing the expression of cell surface molecules involved in immune cell communication. S1P can also regulate the development and function of regulatory T cells, a specialized subset of T cells that play a crucial role in maintaining immune tolerance and preventing excessive immune responses.

Additionally, S1P signaling affects other immune cell populations, including dendritic cells (Zehra Okus et al., 2022), macrophages (Gong et al., 2023), and natural killer cells (Fettel et al., 2019). S1P can modulate the maturation, migration, and cytokine production of these cells, thereby influencing their ability to initiate and regulate immune responses (Fettel et al., 2019). Understanding the precise mechanisms underlying S1P signaling in immune responses is an active area of research. Further studies are needed to elucidate the downstream signaling pathways activated by S1P receptors and their interplay with other immune signaling molecules. Additionally, investigating the therapeutic potential of targeting the S1P signaling pathway in immune-related disorders, such as autoimmune diseases and inflammatory conditions, holds promise for the development of novel treatment strategies.

In conclusion, the S1P signaling pathway exerts profound effects on immune responses by regulating lymphocyte trafficking, immune cell migration, and immune cell function. Continued research in this field will contribute to our understanding of immune regulation and potentially lead to new therapeutic interventions for immune-related disorders.

## 5 Role of S1P in the central nervous system

Ceramide is a central hub for sphingolipid metabolism, and it could be catabolized and phosphorylated into S1P by a family of ceramidases which differ in their pH optimum and subcellular localization (Don-Doncow et al., 2019). In adult human brain, ceramidase is a primary glycoprotein and is primarily expressed in neurons (Don-Doncow et al., 2019). Interestingly, all S1PRs except S1PR4 are expressed in the CNS: oligodendrocytes mainly express S1PR1 and S1PR5, astrocytes S1PR1 and S1PR3, while microglia express S1PR1-S1PR3. A study conducted by Mizugishi group (Mizugishi et al., 2005) consistently showed that mice lacking both Sphk1 and Sphk2 had severe abnormalities in neurogenesis and angiogenesis, resulting in impaired brain development. S1P signaling is crucial during development of the nervous system and in neural progenitor cells by signaling a family of G protein-coupled receptors (Callihan et al., 2018). Based on all of this discussion, S1P is not only involved in vascularization and organ development in the periphery, but also essential for development of the brain (Mizugishi et al., 2005). Furthermore, S1P has anti-apoptotic and pro-growth effects among neural development and mediated by signaling from the S1P1 receptor (Don-Doncow et al., 2019). The activation of S1PRs via S1P contributes to a serious of physiologic processes which involved in neuronal plasticity including myelination, neurogenesis and neuroprotection (Chatzikonstantinou et al., 2021). S1P is more effective than fibroblast growth factor (FGF) in stimulating neurogenesis. Meanwhile, S1P mainly participate in the adjustion of growth cone formation, neurite extension and retraction by binding to S1PRs. In neurons, S1PR1 expression can enhance the neurite extension, while S1PR2 and S1PR5 inhibits neurite extension. Perhaps most interesting of all, S1P has a dual action because of the role of S1P in oligodendrocyte morphology. It enhances retraction function by the regulation of S1PR5, while it promotes process extension via the modulation of S1PR1. In addition, S1P can also act as a chemical attractant to promote the migration of neural stem and progenitor cells (NSPC) to the site of injury and promote the translocation of NSPC to the destination. However, the activation of S1PR5 on oligodendrocytes inhibits their migration. According to the above discussion, S1P can regulate the movement of NSPC and participate in the growth and development of central nervous system cells.

### 5.1 Implication for the role of S1P in brain-derived neurotrophic factors

Brain-derived neurotrophic growth factor (BDNF), one of the most important members of the neurotrophic factor family, was first isolated in 1982 from pig brain by German neurobiologist BARDE (Sandhya et al., 2013). BDNF exists as a homodimer and synthesized as a 32 kDa precursor molecule pro-BDNF, which is post-translationally cleaved to generate the mature and biologically active form of BDNF (m-BDNF, ∼13 kDa) (Barreda Tomás et al., 2020). At present, the biological activity of pro-BDNF is still controversial, but it is certain that m-BDNF plays an important role in maintaining the physiological function of the nervous system. There is a dynamic balance between different forms of BDNF, and the ratio of pro-BDNF to m-BDNF is different in specific stages and regions of brain development. During early development, the ratio of pro-BDNF to m-BDNF is considered to be an important factor in regulating brain function, while m-BDNF plays a crucial role in neuroprotection and synaptic plasticity after maturation. More to the point, BDNF is the second neurotrophin after nerve growth factor (NGF), and is critical for neuronal growth, survival of neurons and phenotypic expression of neuronal cells (Yoshii and Constantine-Paton, 2010). It is regarded as a general modulator of neurotransmitter release, including the release of γ-aminobutyric acid (GABA) which is the primary inhibitory neurotransmitter in the CNS.

BDNF has two binding receptors, high affinity receptor tyrosine kinase receptor B (tyrosine kinase receptor B) and low affinity neurotrophin receptor (p75 nerve growth factor receptor, p75 NTR). After binding with high-affinity tyrosine kinase receptor, tropomyosin-related kinase B (TrkB), receptor dimerization and intracellular tyrosine residue autophosphorylation were activated. Phosphorylated TrkB can promote neuronal survival, increase synaptic plasticity, and play a neurotrophic role via phospholipase C-γ/protein kinase C (PLC-γ/PKC), mitogen-activated protein kinase (MAPK), RAS/ERK, JAK/STAT and phosphatidylinositol-3-kinase (PI3K)/protein kinase B (PKB/AKT) pathways (Sandhya et al., 2013). PI3K/AKT signaling, stimulated by BDNF is crucial for proliferation, protection and survival of neuronal cells (Yamada et al., 2001; Sandhya et al., 2013). BDNF stimulation of ERK5/MEF pathway is essential for neuronal survival (Shalizi et al., 2003) and various cellular processes including growth (Sugimoto et al., 2001), differentiation (Yin et al., 2010), protection of neuronal cells (Szatmari et al., 2007). The activation of PLC also leads to intracellular calcium release and CREB phosphorylation (Finkbeiner et al., 1997), neuronal migration and increase synaptic plasticity (Zuccato et al., 2001). BDNF also binds to p75 NTR, albeit with a low affinity, and participate in the induction of some cell apoptosis and pathological injury process. Among the neurotrophins, BDNF has been become as a major regulator of synaptic plasticity, survival of neurons and neuronal differentiation, and also as a novel and valuable target for drug development in neurological disorders.

Sphingosine-1-phosphate enhances the expression of neurofilament and promote neuronal survival which induced by NGF, while inhibit SphK activity to eliminate neuronal cell differentiation (Edsall et al., 1997). The axon elongation of neuronal cells and dorsal root ganglion neuron induced by NGF depends on the activity of S1P (Toman et al., 2004). While, NGF can promote axon elongation to activate S1P through stimulates Sphk1 activity through TrkA receptor. S1P induced the expression and release of BDNF in neuronal cells while promoting the growth and proliferation of astrocytes (Yamagata et al., 2003). In addition, BDNF increases the expression of SphK1 in neuroblastoma cell lines, leading to the production and secretion of S1P (Murakami et al., 2011). Generally, it can be seen that there may be interaction and expression between S1P and BDNF. Upon binding to GPI-anchored coreceptors known as glial cell line-derived neurotrophic factor (GDNF) family alpha (GFRα), GDNF triggers the activation of the RET receptor tyrosine kinase, subsequently initiating the activation of various downstream signaling pathways (Murakami et al., 2011). The GDNF receptor RET induces the expression of the SPHK1 gene, and there are indications that SPHK1 plays a part in GDNF-induced differentiation. Therefore, the SPHK/S1P signaling pathways activated by neurotrophic factors play a crucial role in neural differentiation (Murakami et al., 2007). Elucidation of these mechanisms might also be helpful for the understanding of the clinical PD.

### 5.2 Implication for the role of S1P in neuroregulation

In the CNS, S1P can regulate the release of neurotransmitters and the excitability of neurons, and this regulation may be achieved through blinding to S1PR. Interestingly, S1P facilitated glutamate secretion in hippocampal neurons in a dose-dependent manner, indicating its potential involvement in the regulation of synaptic transmission by S1P/S1PR3 pathway (Kanno and Nishizaki, 2011; Sukocheva, 2018), while inhibited glutamatergic nerve transmission through S1PR1 (Sim-Selley et al., 2009). S1PR2-deficient mice showed a significant increase in excitatory postsynaptic enhancement, leading to spontaneous seizures in these animals. Consistent with these observations, S1P can enhance the excitability of small-diameter sensory neurons (Li et al., 2015). According to the above, the all evidence suggests that S1P may also be involved in the regulation of neuronal excitability.

### 5.3 Implication for the role of S1P in neuroprotection

Sphingosine-1-phosphate plays an important role in the brain as a neuromodulator and a neuroprotector. By far the greatest risk factor for neurodegenerative diseases including PD is aging, and mitochondria have been thought play an important role in aging by mitochondrial dysfunction, oxidative stress, and cell apoptosis. Mitochondria are double-membrane organelles responsible for oxidative energy metabolism in eukaryotic cells. In addition, it plays an important role in the maintenance of Ca^2+^ homeostasis (Witte et al., 2014; Figure 2; Table 1) and participates in the control generation of reactive oxygen species (ROS). SphK2, expressed in nucleus and mitochondria, is responsible for the local production of S1P in the mitochondria (Strub et al., 2011). Interestingly, S1P produced in the mitochondria own high affinity and specificity to prohibitin 2 (PHB2) which is a key factor in the normal development, morphogenesis and function of mitochondria as a highly conserved protein (Aoki et al., 2016). In mitochondria from SphK2-null mice, the interaction between PHB2 and subunit (I and IV) of cytochrome-c oxidase is greatly reduced (Strub et al., 2011). Most likely, S1P is necessary for the biogenesis of autophagosomal precursors via mitochondrial DNA replication and transcription (Shen et al., 2014).

Oxidative stress can activate apoptosis pathway by mitochondrial-dependent and non-mitochondrial-dependent ways. However, S1P inhibits the apoptosis pathway activated by mitochondrial-dependent method. Initial evidence implicating the effect of oxidative stress on cell fate is bidirectional. It will cause the activation of sphingomyelinase and shift the “sphingolipid rheostat” toward ceramide accumulation when the production rate of ROS exceeds the oxidation capacity of cells, thus triggering cell apoptosis (Petrache and Berdyshev, 2016). On the other hand, SphK1 can be activated by low-level oxidative stress which upregulated the level of S1P, thus facilitating cell survival (Motyl et al., 2021). S1P play a crucial role in cellular damage induced by oxidative stress and validated via human dopaminergic neuronal cell (SH-SY5Y) (Pyszko and Strosznajder, 2014). More interestingly, only S1P produced by SphK2 can cause cell apoptosis, suggesting the biological function of S1P in subcellular localization (Hao et al., 2020). Furthermore, the generate of ceramide mediated sphingomyelinase can be inhibited by S1P (Czubowicz et al., 2019).

Sphingosine-1-phosphate signal pathway can regulate autophagy which plays a crucial role in the cells survival such as neurons (Mitroi et al., 2017). Bioactive sphingolipids, such as ceramide and S1P, play a vital role in cellular apoptosis, senescence and growth arrest. Initial evidence implicating that the dynamic equilibrium between the formation, catalyzed by SphKs, and degradation, catalyzed by S1P phosphatases (SGPPs) and sphingosine phosphate lyase 1 (SGPL1), of S1P, is crucial to S1P-related autophagic processes (Hao et al., 2020). It is illustrated that ceramide can help to facilitate autophagy-associated cell death and can be blocked by S1P by transactivation of mTOR via S1PR1 and S1PR3 (Taniguchi et al., 2012). Also, similar results have been shown in SphK2, S1P-induced apoptosis can be prevented through the pretreatment with SphK2 inhibitors (Hao et al., 2020). Therefore, S1P-induced autophagy in intracellular can protect cells from death during nutrient deficiency and has the characteristics of apoptosis by reabsorption function (Lavieu et al., 2006). Supporting this concept, S1P-induced autophagy in intracellular is a cellular defense mechanism, which play a vital role in cell survival.

## 6 The relationship between S1P and Parkinson’s disease

### 6.1 Gene expression profiles and data preprocessing in the metabolism of S1P

**FIGURE 3:**
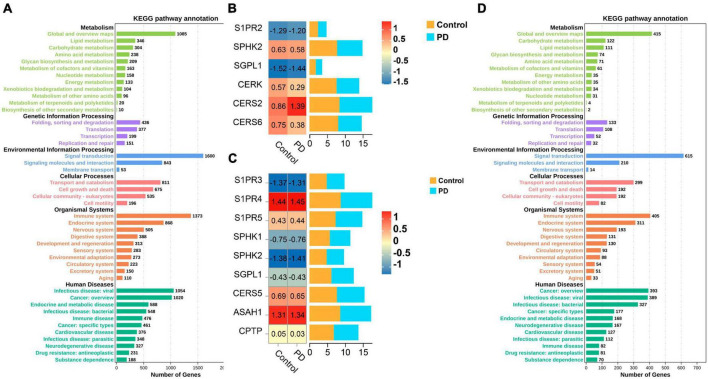
**(A,D)** The metabolic pathway (KEGG) enrichment analysis based on the GEO database which related with the development of PD; **(B,C)** Dynamic heat maps and bar charts provide a visualization of the differentially expressed genes which related with S1P metabolism between healthy and PD patients.

The gene expression data was obtained from the Gene Expression Omnibus (GEO) database, comprising GSE8397 and GSE99039. One-way ANOVA test was performed on all selected samples to filter genes with a *p*-value less than 0.05. Then, the differentially expressed genes were converted to their gene IDs using omicshare gene ID tool. Additionally, the genes obtained will be subjected to metabolic pathway (KEGG) enrichment analysis by omicshare KEGG enrichment analysis tool. In our findings, we discovered a substantial connection between the development of PD and the metabolic processes of lipids (Figures 3A, D). What’s even more astonishing is the finding of an increased quantity of genes exhibiting a heightened association with S1P metabolism among differentially expressed genes. Dynamic heat maps and bar charts provide a visualization of the differentially expressed genes which related with S1P metabolism between healthy and PD patients (Figures 3B, C).

### 6.2 Crosstalk between S1P and the pathogenesis of PD

Parkinson’s disease is a neurodegenerative disease and the main pathological feature is the progressive degeneration or loss of dopaminergic neurons in the substantia nigra pars compacta (SNpc), which leads a lack of dopamine in the striatum (Samii et al., 2004). In addition to the loss of nigrostriatal dopamine neurons, accumulation of alpha-synuclein protein (α-syn, targeting dopamine neurons in the midbrain) in the form of Lewy bodies (LBs), leading the ability to synthesize dopamine reduce, is an important feature in PD (Su et al., 2021; Mahapatra et al., 2022). The function of S1P in the degradation of α-syn is to facilitate the breakdown and clearance of α-syn protein, potentially preventing its accumulation and the formation of aggregates through activation of the mTOR pathway (Mitroi et al., 2017). Autophagic flux is blocked at initial stages upon SGPL1 deficiency (Zhang et al., 2017). On the basis of these results, the balance between the acetylcholine loop and dopamine system is broken, leading to the motor symptoms and non-motor symptoms seen in patients (West et al., 2022). At present, the etiology and pathogenesis of PD is only partly understood. Notably, several observations suggest that oxidative stress, mitochondrial dysfunction, inflammation and protein misfolding are involved in the pathogenesis of PD (Wang et al., 2013; Li et al., 2021), which can be reversed by FTY720 (a structural sphingosine analog and S1PR modulator) (Zhang and Wang, 2020).

Increased oxidative stress, induced by the high basal rates of oxidative phosphorylation leading to the mitochondrial dysfunction in nigral neurons. The relationship between mitochondrial dysfunction and PD was first found by Schapira et al. (1990). Interestingly, decreased complex I activity in PD substantia nigra tissue appears to be specific to PD (Macdonald et al., 2018). Complex I, the largest mitochondrial complex, is a type-I NADH dehydrogenase which contain at least 44 subunits (Macdonald et al., 2018). The complex couples the transfer of two electrons from NADH to ubiquinone and translocates of four protons across the membrane (Schulte et al., 2019). Collectively, impairment of mitochondrial function result in decreased ATP levels, increased levels of reactive oxygen species (ROS), impaired calcium buffering capacity, elevated apoptosis, impaired mitochondrial membrane potential (MMP) and impaired calcium buffering capacity (Wang et al., 2019) which was related with age-associated muscle dysfunction (Gill et al., 2019). One particularly intriguing observation is that the activity of complex (I, II, III, or IV) remained unchanged before and after treatment (Shults et al., 1995). At present, data from the literature show that not only are mitochondrial deficiencies present before drug treatment (Punzi et al., 2018), but also that current drug therapy do not inhibit mitochondrial damage and resolve abnormal mitochondrial functions (Murthy et al., 2022). These findings suggest that targeting the S1P pathway might be therapeutically important for PD patients through regulating mitochondrial function.

Within the realm of translational medicine, it has become apparent to us that mitochondrial dysfunctions are the key factors in the mitochondrial biogenesis caused by the transcription-factor dysregulation, leading to the adverse effect on cellular bioenergetics. In particular, peroxisome proliferator-activated receptor-gamma coactivator-1α (PGC-1α), a coactivator of several transcription factors, play an important role in mitochondrial biogenesis (Xu et al., 2023). According to previous research findings, the overexpression of PGC-1α caused dopamine depletion is relative to low expression levels of paired-like homeodomain 3 (PITX3), increased susceptibility to MPTP (Corona and Duchen, 2015; Zhong et al., 2023) and reduced a-synuclein levels, whereas deficiency of PGC-1α is related with the propensity of a-synuclein to oligomerize (Punzi et al., 2018). On a functional site, the expression of PGC-1α reduced by the inhibition of Sphk2, down-regulated in the brain of PD patients (Sivasubramanian et al., 2015). Overall, therefore S1P/PGC-1α appears to play a neuroprotective role in PD. S1P modulates mitochondrial dysfunction by interacted with mitochondrial proteins, including Bak protein, and is also relevant to the release of cytochrome C (Pyszko and Strosznajder, 2014). The electron transport chain in the respiratory chain is blocked because of the deficiency of cytochrome C which is the important part of the mitochondrial respiratory chain, leading to the decrease of ATP synthesis and the excessive production of ROS caused by incomplete oxidation. It is well established that increased levels of mutations and deletions of mitochondrial DNA (mtDNA) are a common cause of PD patients (Siibak et al., 2017). Human mitochondrial DNA, consisting of 16,569 base pairs, is a double stranded molecule that is 15∼16 kb in size (Sun et al., 2018). MtDNA encodes for 13 proteins along with the 22 tRNAs and 2 rRNA (Hauser and Hastings, 2013). Another intriguing possibility that could underlie the mtDNA deletions are specific to nigral neurons which increase their contribute to in PD (Hauser and Hastings, 2013). However, the usage of S1P could obviously upregulate mitochondrial DNA replication and transcription, increase mitochondrial mass, and elevated adenosine triphosphate synthesis (Shen et al., 2014), leading to the improvement of PD patients.

Increasing evidence suggests that the accumulation of misfolded α-syn play an important role in neurodegenerative diseases including PD. To be gratified, the secreted of α-syn can be facilitated by inhibited SphKs, while α-syn can reduce the activity and protein expression level of SphK1, leading to molecular structure changes and cell death (Murphy et al., 2014). There is also some form of antagonism between S1P and its downstream proteins by α-syn. α-Syn can block the activity of essential proteins in downstream signal transduction of S1PR1, including BDNF/Akt kinase signal pathway (Safarian et al., 2015; Kang et al., 2017). In addition, the interaction between α-syn and S1P also influence mitochondrial homeostasis and protect DA neurons in MPTP/MPP + model.

### 6.3 S1P receptor modulators and the functions of it in PD

**FIGURE 4:**
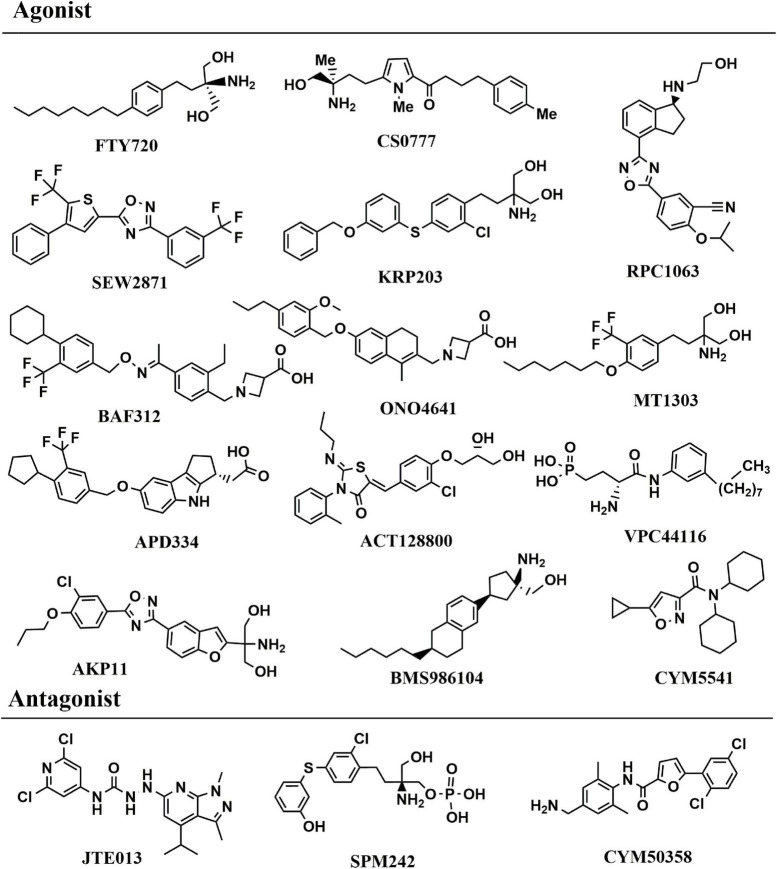
Chemical structure of S1PRs agonist and antagonist.

The most direct evidence in Schwedhelm et al. (2021) research, the level of serum S1P in patients with PD was lower than that in healthy controls, and decreased gradually with the increase of the severity of the disease by measuring the level of serum S1P in human body for the first time. This study demonstrated the correlation between S1P level and the severity of PD patients in human body. At present, S1P receptor modulators are mainly focused on FTY720, SEW2871 and CS0777, etc. FTY720 (2-Amino-2-[2-(4-octylphenyl) ethyl]propane-1,3-diol hydrochloride, Fingolimod, chemical structure in Figure 4), was the first S1P receptor modulator to be approved by United States Food and Drug Administration in 2010 for the treatment of multiple sclerosis, followed by siponimod, ozanimod, and ponesimod (Figure 4; Cohan et al., 2022). Emerging evidence have illustrated that Fingolimod can exert neuroprotective action to battle against PD (Pépin et al., 2020). The structure of fingolimod is similar to sphingosine and phosphorylated to fingolimod-P (an S1P analog) by SphK1/2 (Roy et al., 2021). Similar to S1P, fingolimod-P can bind to the S1P receptor, leading to the S1P receptor degraded (Roy et al., 2021). One of the most interesting findings, fingolimod promotes the levels of BDNF which is a crucial neurotrophic factor for dopaminergic neurons (Vidal-Martinez et al., 2019). In association with PD, BDNF is a major neurotrophin for SNc dopaminergic neurons, thereby using an agent to increase BDNF expression is a potential method is a promising PD therapeutic (Vidal-Martinez et al., 2019). Meanwhile, fingolimod significantly improved behavioral deficits, decreased the loss of dopaminergic neurons and promoted dopamine release by neuroinflammation pathway (Yao et al., 2019). Similar to the FTY720, CS0777 (1-{5-[(3R)-3-amino-4-hydroxy -3-methylbutyl]-1-methyl-1H-pyrrol-2-yl}-4-(4-methylphenyl) butan-1-one), a selective S1P1 modulator, is phosphorylated to an active S1P analog (Yao et al., 2019) (CS0777-P, Figure 4). SEW2871 (5-(4-phenyl-5-trifluoromethylthiophen-2-yl)-3-(3-trifluorometh ylphenyl)-1,2,4-oxadiazole, Figure 4), a selective agonist to subtype 1 of S1PRs, can protect dopaminergic neurons and improve motor deficits in PD (St-Cyr Giguère et al., 2017). Naturally, the other S1P modulator and the functions in PD were summarized in Table 2. Compared to FTY720, most other S1PR modulators provide safety advantages and fewer side effects because of the selective mode blinding to S1PR. However, the pharmacological and rational theory for the drug development of PD remain inadequate. Up to now, there is no targets of S1P reported in PD. Meanwhile it also shows that the S1P receptor modulators are an ideal therapeutic agent for PD.

**TABLE 2:** Specificity of S1PR modulators and the functions in PD.

S1P receptor modulator	Receptor	Regulatory effect on S1P	Active form	Binding form to S1P	Generic name	Function in PD	References
FTY720	S1PR1, S1PR3, S1PR4, S1PR5	Agonist	FTY720 phosphate	Non-selective	2-Amino-2-[2-(4-octylphenyl)ethyl]propane-1,3-diol hydrochloride	Increase the level of BDNF; Own neuroprotective effects; Protect against neuroinflammatory cell death; Stimulates PP2A to enhance the activity of α-syn; Reduce PD progression by inhibiting NLRP3 inflammasome activation; Abolish the loss of TH immunoreactivity.	Nishi et al., 2011; Ren et al., 2017; Motyl et al., 2018; Vidal-Martinez et al., 2019; Mirzaei et al., 2022.
CS0777	S1PR1	Agonist	CS0777 phosphate	Selective	1b{5b[(3R)b3baminob4bhydroxyb3bmethylbutyl] b1bmethylb1Hbpyrrolb2byl}b4b(4bmethylphenyl) b1bbutanone	–	Yonesu et al., 2011; Radeke et al., 2020.
SEW2871	S1PR1	Agonist	SEW2871 phosphate	Selective	5-(4-phenyl-5-trifluoromethylthiophen-2-yl)-3-(3-trifluoromethylphenyl)-1,2,4-oxadiazole	Increase the level of BDNF; Protect against neuroinflammatory cell death; Protect the formation of a-syn aggregates, dopaminergic neurons and motor deficits.	Pépin et al., 2020.
KRP203	S1PR1	Agonist	KRP203 phosphate	Selective	2baminob2b[2b[2bchlorob4b[[3b(phenylmethoxy) phenyl]thio]phenyl]ethyl]b1,3bpropanediolhydroc hloride	–	Khattar et al., 2013; Radeke et al., 2020; McGinley and Cohen, 2021; Dertschnig et al., 2023.
BAF312	S1PR1, S1PR5	Agonist	–	Selective	1-[[4-[(E)-N-[[4-cyclohexyl-3-(trifluorometh yl) phenyl]methoxy]- C-methylcar bonimidoyl]-2-ethyl phenyl]methyl]azetidine-3-carboxylic acid	Regulate microglial activity and BBB stability.	Spampinato et al., 2022.
RPC1063	S1PR1, S1PR5	Agonist	–	Selective	5-[3-[(1S)-1-(2-hydroxyethylamino)-2,3-dihydro-1H-inden-4-yl]-1,2,4-oxadiazol-5-yl]-2-propan-2-yloxybenzonitrile	Reduces the destruction of the BBB; Own neuroprotective effects;	Scott et al., 2016; Wang et al., 2022.
ONO4641	S1PR1, S1PR5	Agonist	ONO4641	Selective	1b({6b[(2bmethoxyb4bpropylbenzyl)oxy] b1bmethylb3,4bdihydronaphthalenb2byl}methyl) azetidineb3bcarboxylic acid	–	Komiya et al., 2013.
APD334	S1PR1, S1PR4, S1PR5	Agonist	–	Selective	(R)b2b(7b(4bcyclopentylb3b(trifluoromethyl) benzyloxy) b1,2,3,4b tetrahydrocyclopenta[b]indolb3b yl)acetic acid	–	Sandborn et al., 2020; Vermeire et al., 2021; Wils and Peyrin-Biroulet, 2023.
ACT128800	S1PR1	Agonist	ACT128800	Selective	(Z,Z)-5-[3-chloro-4-(2R)-2,3-dihydroxy-propoxy)-benzylidene]-2-propylimino-3-o-tolylthiazolidin-4-one	–	D’Ambrosio et al., 2016; Ruggieri et al., 2022.
MT1303	S1PR1, S1PR5	Agonist	MT1303 phosphate	Selective	2-amino-2-{2-[4-(heptyloxy)-3-(trifluorome thyl) phenyl]ethyl}prop an-1,3- diolhyd rochloride	–	Sugahara et al., 2017; Sugahara et al., 2019; Tanaka et al., 2020.
VPC44116	S1PR1, S1PR3	Agonist	VPC44116	Selective	[(3R)-3-amino-4-(3-octylanilino)-4-oxobutyl] phosphonic acid	Promote glutamatergic neurotransmission as determined by electrophysiological recordings in cortical neurons.	Wamhoff et al., 2008; Sim-Selley et al., 2009.
AKP-11	S1PR1	Agonist	–	Selective	2-amino-2-[5-[5-(3-chloro-4-propoxyphenyl)-1,2,4-oxadiazol-3-yl]-1-benzofuran-2-yl]propane-1,3-diol	–	Samuvel et al., 2015.
BMS986104	S1PR1	Agonist	BMS986104 phosphate	Selective	[(1R,3S)-1-amino-3-[(6R)-6-hexyl-5,6,7,8-tetrahydronaphthalen-2-yl]cyclopentyl]methanol	–	Dhar et al., 2016; Yang et al., 2016; Yuan et al., 2018; Gilmore et al., 2019.
JTE013	S1PR2	Antagonist	–	Selective	N-(2,6-dichloro-4-pyridinyl)-2-[1,3-dimethyl-4-(1-methylethyl)-1H-pyrazolo[3,4-b]pyridin-6-yl]-hydrazinecarboxamide	–	Liu et al., 2021; Zhou et al., 2022; Lory et al., 2023.
CYM5541	S1PR3	Agonist	–	Selective	N,N-dicyclohexyl-5-cyclopropyl-1,2-oxazole-3-carboxamide	–	Wang et al., 2018; Paradiso et al., 2021.
SPM242	S1PR3	Antagonist	SPM242	Selective	[(2S)-2-amino-4-[2-chloro-4-(3-hydroxyphenyl)sulfanylphenyl]-2-(hydroxymethyl)butyl] dihydrogen phosphate	–	Jo et al., 2012.
CYM50358	S1PR4	Antagonist	–	Selective	N-[4-(aminomethyl)-2,6-dimethylphenyl]-5-(2,5-dichlorophenyl)furan-2-carboxamide	–	Guerrero et al., 2011; Jeon et al., 2021.

## 7 Conclusion

Plasma S1P, which can improve PD development and progression, levels are reduced in PD patients compared to healthy controls. Meanwhile, S1P can protect against dopamine depletion, neuroinflammation, and PD-associated symptoms by uncoupling the S1P1R from Gi-protein. Most impressively, some studies have illustrated that S1P might regulate microglial phagocytosis. Thus, S1P receptor modulators are promising target in the therapy of PD. Hence, developing further understanding about the role of S1P in PD development and progression is of great significance and urgency to promote the timely and accurately diagnosis of PD. The drug development which focused on the S1P and S1P receptor modulators have implications for improving the diagnosis and therapy of the PD patients.

## Author contributions

WW: Funding acquisition, Writing—original draft, Writing—review and editing. YZ: Conceptualization, Supervision, Writing—original draft, Writing—review and editing. GZ: Conceptualization, Funding acquisition, Supervision, Visualization, Writing—original draft, Writing—review and editing.
